# Documents Publication Evolution (1990–2022) Related to Physical Activity and Healthy Habits, a Bibliometric Review

**DOI:** 10.3390/healthcare11121669

**Published:** 2023-06-06

**Authors:** Víctor Hernández-Beltrán, Mário C. Espada, Fernando J. Santos, Cátia C. Ferreira, José M. Gamonales

**Affiliations:** 1 Optimization of Training and Sports Performance Research Group, Faculty of Sport Science, University of Extremadura, 10005 Cáceres, Spain; vhernandpw@alumnos.unex.es (V.H.-B.); catia.ferreira@ese.ips.pt (C.C.F.); martingamonales@unex.es (J.M.G.); 2 Instituto Politécnico de Setúbal, Escola Superior de Educação, CIEF, CDP2T, 2914-504 Setúbal, Portugal; fernando.santos@ese.ips.pt; 3 Life Quality Research Centre (CIEQV-Leiria), 2040-413 Rio Maior, Portugal; 4 CIPER, Faculdade de Motricidade Humana, Universidade de Lisboa, 1499-002 Lisboa, Portugal; 5 Faculdade de Motricidade Humana, Universidade de Lisboa, 1499-002 Lisboa, Portugal; 6 Faculty of Health Sciences, University of Francisco de Vitoria, 28223 Madrid, Spain

**Keywords:** scientometrics, physical fitness, exercise, quality of life, obesity

## Abstract

This research aims to provide an overview of the growing number of scientific literature publications related to Physical Activity and Healthy Habits. A bibliometric analysis between 1990 and 2022 in the Web of Science was carried out, following the bibliometric analysis law, using Microsoft Excel and VosViewer Software for analysis and data processing. A total of 276 documents (262 primary studies and 14 revisions) related to the topic under study were located. The results reveal an exponential growth of scientific production by 48% between 2006 and 2022. Public Environmental Occupational Health, Kaprio, J., and the USA were the knowledge field, author, and country most productive, respectively. A great thematic diversity was found related to the most used keywords by the authors, such as “physical activity”, “health habits”, “exercise”, and “obesity”. Thus, the research related to this theme is really in an exponential phase, with great interest in the importance of physical activity and healthy habits, implying practical decisions in policies to develop programs to promote physical activity and healthy habits.

## 1. Introduction

Currently, there has been an increase in people with obesity due to physical inactivity and lack of healthy habits, with the prospect that by the year 2030, 57.8% of the world population will be overweight or obese [1,2,3]. The data related to physical inactivity are accentuated in women [4], and they verify that it is accentuated with age [5,6]. Estimates of physical activity achievement indicate that most people are not very active. In response to these distressing statistics, numerous investigations are being carried out with intervention programs for people of different ages [7,8,9]. In other words, the programs applied in the educational stages have been shown to demonstrate greater effectiveness at the time of modifying eating habits and produce an increase in the level of physical activity, with the aim of avoiding a sedentary lifestyle and the risk of illness related to obesity [10]. Therefore, there is a growing decrease in behaviors that meet healthy lifestyles [11,12].

In the scientific literature, there are investigations that show how the lack of physical activity, the choice of unhealthy foods, and contagion are factors that influence different chronic diseases [13,14]. Chronic illnesses are a global challenge and require medical attention in populations in countries with low and medium incomes as a consequence of changes in lifestyles [14,15]. Because of more sedentary lifestyles and maladjusted eating habits, obesity has a higher prevalence, being an important factor in chronic diseases and having implications for “insulin resistance” or “metabolic” syndrome, hypertension, diabetes mellitus type II, etc., and a higher risk of cardiovascular diseases [16]. For this reason, the adoption of healthy lifestyles should be encouraged, through the practice of physical activity and healthy habits.. Therefore, physical activity and healthy habits must be promoted to reduce associated diseases.

There are recent investigations linked to the identification of optimal patterns of physical sports practice with the aim of having good health [17]. Furthermore, there are studies related to the association between healthy habits and physical activity in young people [10], adults, and older people [18,19]. Likewise, there are studies related to diet and physical activity as factors that directly influence health [20,21]. Therefore, physical activity contributes to the prevention of illnesses such as cardiovascular diseases, type II diabetes mellitus, overweight and obesity, as well as colon cancer, depression, and anxiety, among others [16,21]. In addition, physical activity contributes to the improvement of the psychological state of people [5,21], and constitutes one of the behaviors that promote a healthy lifestyle [22,23]. In this way, and in order to keep people in a regular practice of physical activity, it is essential that it is associated, whether formal or informal, with fun, satisfaction, and pleasure.

Within unhealthy habits, there is the consumption of alcohol and tobacco [24,25,26], as well as the consumption of drugs [27]. Another negative factor is the use of new technologies that negatively influence the physical activity level [10,28,29] since many of the hours of their free time are occupied with television (TV), mobile phones, or video games. Therefore, technological advances have provided people with a new way of life [30] and have even harmed healthy lifestyles. These factors are linked to a sedentary lifestyle and an increase in obesity [31]. However, at present, there is much knowledge and many learning environments that allow and favor the practice of physical activity, for example, virtual works [32], natural [33], and urban-green-space environments [34].

After the literature review, it is necessary to expand the knowledge related to physical activity and healthy habits, and the importance of healthcare. For this reason, the aim of this study is to carry out a bibliometric review regarding the terms “Health habits” and “Physical activity”, with the aim of assessing the evolution of the documents related to these concepts as fundamental tools to improve the healthy lifestyle of people, from their origin until the year 2022.

## 2. Materials and Method

### 2.1. Design

The present study fits within research called “ex post facto retrospective” [35] because a bibliometric analysis was performed taking as reference the bases of bibliometric [36]. In addition, carrying out a study and bibliometric analysis of the identified documents will allow the evolution and trend of the number of publications in relation to a thematic line to be understood [37]. In the same way, a series of elements or factors are identified, providing knowledge and favoring the understanding of the contents [38].

### 2.2. Data Source

For the search and identification of documents, the Web of Science (WOS) database has been used. With the purpose of obtaining the largest number of documents related to the subject of the study, this database was selected, since it is the one that encompasses the largest number of documents, as it is the most used in the scientific literature for performing two analyses [39,40,41]. This database offers information on journals classified according to their main thematic topics, as well as on their influence in the scientific field [42]. In addition, it allows you to extract data from documents related to title, abstract, year of publication, number of citations received, journal of publication, co-authors, and type of document, among others.

### 2.3. Search Strategy

**Figure 1:**
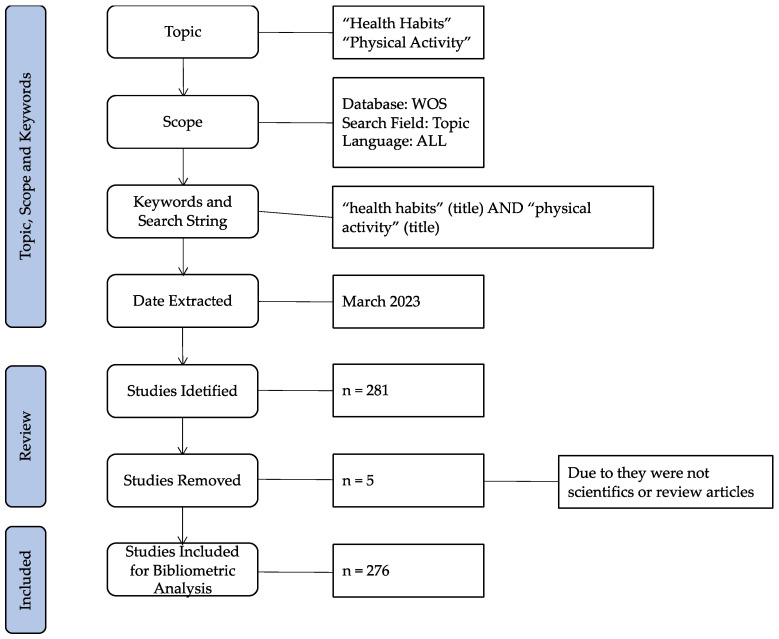
Flow chart of the search.

The search was completed in March 2023. The keywords (“health habits” and “physical activity”) were entered into the database using the “Topic” search filter. Later, the documents were reviewed regarding the inclusion and exclusion criteria established by the researchers: to select only scientific or review articles; to present key terms in the title, abstract, or keywords of the document; and to be related to the research topic. After the revision of the identified documents, and the elimination of the duplicated manuscripts in the process, a total of 276 selected documents were obtained up to 31 December 2022, from the WOS Core Collection. This process of the search and evaluation of the documents was carried out by two researchers to minimize the bias of the results and identify the major number of documents related to the topic. Next, in Figure 1, a flow chart about the search of the documents is shown.

### 2.4. Statistical Analysis

A descriptive analysis was carried out based on the number of publications produced per year, with the aim of assessing the exponential growth, as well as identifying the trend in the increase in the number of studies. This process was performed through the R^2^ coefficient of determination, using DeSolla Price’s law of Exponential Growth of Science [43,44]. Likewise, the law of Lotka was implemented using the Hirsch Index (Index h) [45], with the objective of recognizing those authors with the greatest number of publications, and, with the greatest number of citations [46].

The keywords most employed were selected through the use of Zipf’s law [47]. The data were downloaded in different formats, Excel and plain text, for further analysis using Microsoft Excel^®^ (2006 version; Microsoft Corporation, Redmond, WA, USA) and VOSviewer (Center for Science and Technology Studies, Leiden, The Netherlands) software. For the analysis and creation of figures in VOSviewer, an attraction force (3) and a repulsion force were used (−3).

## 3. Results

### 3.1. Annual Publication Trends

**Figure 2:**
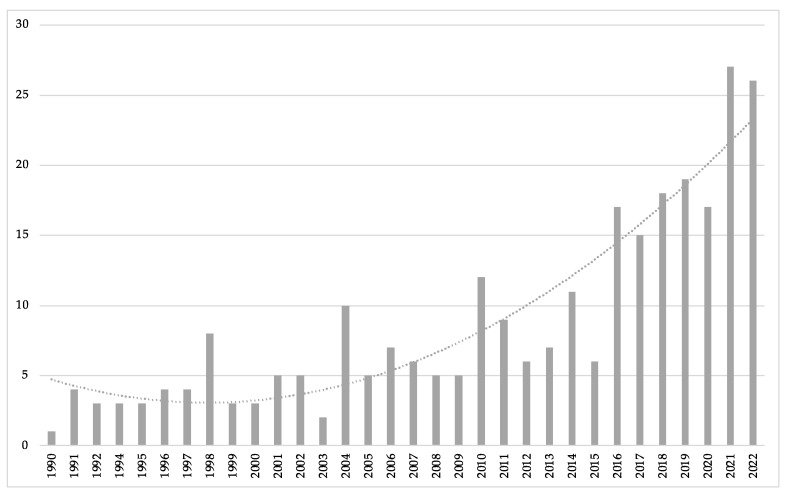
The exponential growth of annual publications on health habits and physical activity.

In the search, a total of 276 documents were located (262 primary studies and 14 revisions) related to the topic under study. Since 1990, there has been a clear continuity and increase in the number of publications, showing exponential growth with an R^2^ value of 48% (Figure 2).

### 3.2. WOS Categories about the Areas of Knowledge

In Table 1, the documents are shown related to the study area/field. It is observed that the main fields present the largest number of documents (Public Environmental Occupational Health, Medicine General Internal, Nutrition Dietetics, and Sport Science). For this reason, the manuscripts are closely related to the study of health, nutrition, and sports, which are mentioned as the main elements for developing healthy lifestyle habits. In addition, these areas of knowledge collect more than 58% of the publications related to the study topic.

### 3.3. H-Index

**Figure 3:**
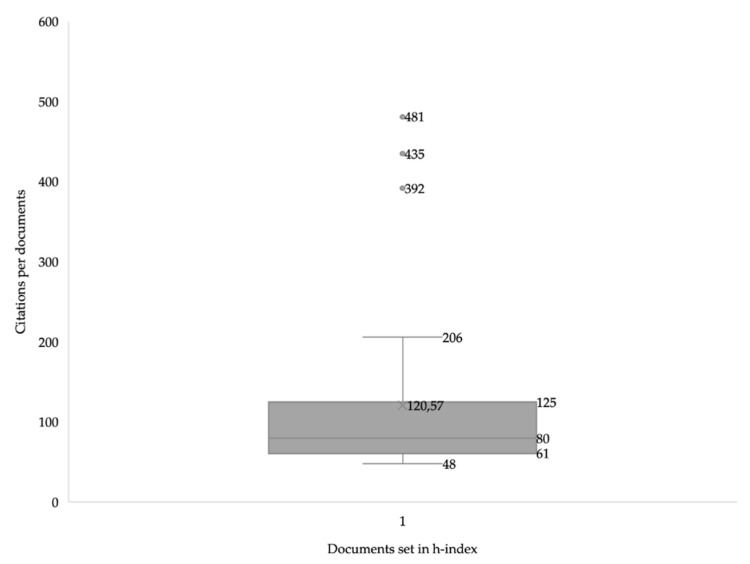
Citations per article on “health habits” and “physical activity”.

After applying the H-index to the different selected documents, we found that in 48 studies a large number of citations (*n* > 48) was recorded. In addition, a range of the number of citations is observed between 48 and 481, with 120 being the average citation number for each study. On the other hand, three of the selected documents present more than 400 citations, Hasler et al. [48], with 481 citations, Kujala et al. [49], with 436 citations, and Vita et al. [50], with 435 citations (Figure 3).

### 3.4. Relationship between the Co-Authors of the Publications

**Figure 4:**
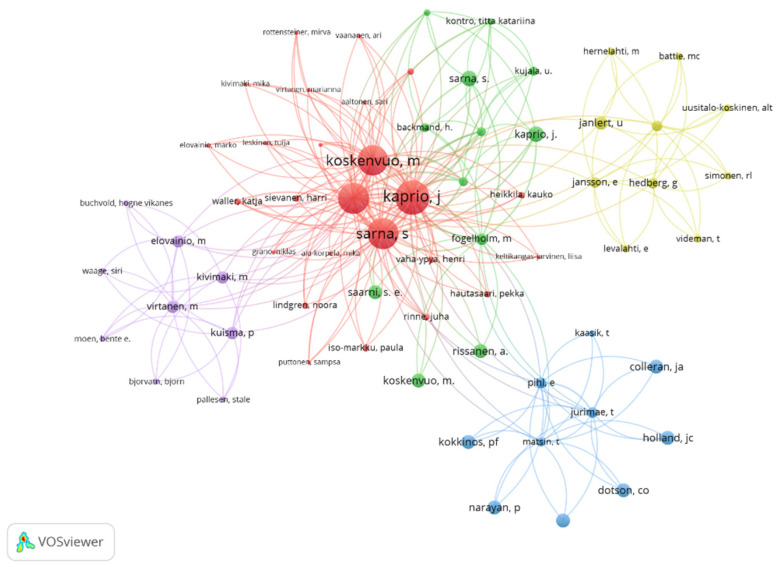
Relation of co-authors based on the number of citations.

In Figure 4, the relationships between the different co-authors of the selected documents are shown. Five different work groups are observed, with the most predominant network formed by the authors: “Koskenvou”, “Kaprio”, and “Sarna”. In addition, these are the researchers who present the greatest number of citations in relation to physical activity and healthy habits.

### 3.5. Publications in Function of the Countries

**Figure 5:**
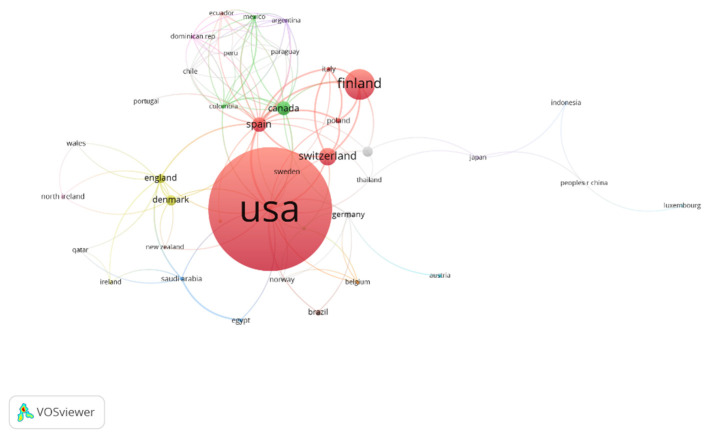
List of countries based on the number of citations received.

The studies reported in the publications that related to “physical activity” and “healthy habits” were carried out in different countries or regions, due to the importance of their dissemination and analysis. In Figure 5, the existing relationships between the different countries involved can be observed. The countries of the United States of America (USA), Spain, Switzerland, and Finland are the territories that present a greater number of interactions. In the same way, the USA and Finland are among the countries that receive the highest number of citations.

**Figure 6:**
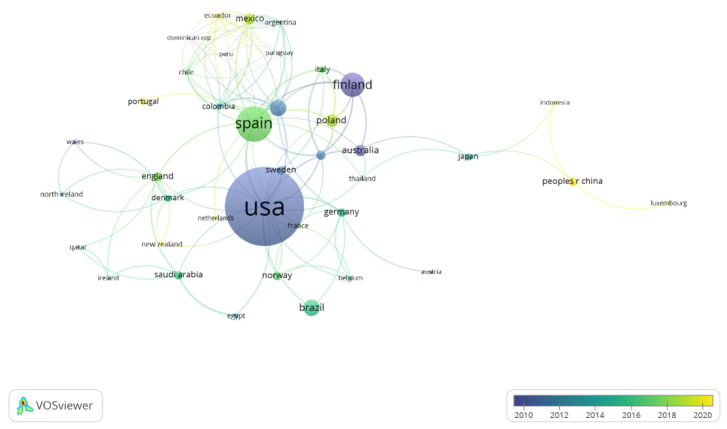
Relation of the countries according to the temporality of publication.

On the contrary, depending on the year of publication (Figure 6), it is observed that the USA and Finland are those regions with an average year of publication that is relatively old. For this reason, they present a greater number of citations than the rest of the countries. In the same way, Spain and Poland also have a higher number of publications, as well as the most recent ones. On the contrary, it is observed that Luxembourg, Indonesia, and Portugal are regions that are increasing their number of publications, due to their current interest in the thematic object of study. In addition, these countries are the ones with the greatest number of current publications.

### 3.6. Author’s Keywords

**Figure 7:**
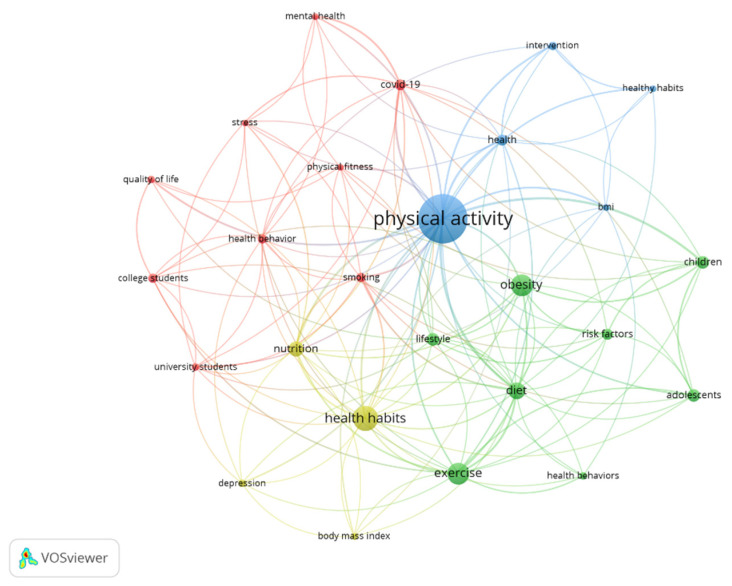
Relation of the keywords.

A total of 695 keywords established by the authors were identified. After the application of the Zipf law, for the analysis of the key terms, a total of 26 words were obtained with an occurrence greater than or equal to six. The most frequently used terms were “physical activity” (59), “health habits” (27), “exercise” (23), and “obesity” (23). Figure 7 shows the interaction between the different keywords used by the authors in their different documents.

**Figure 8:**
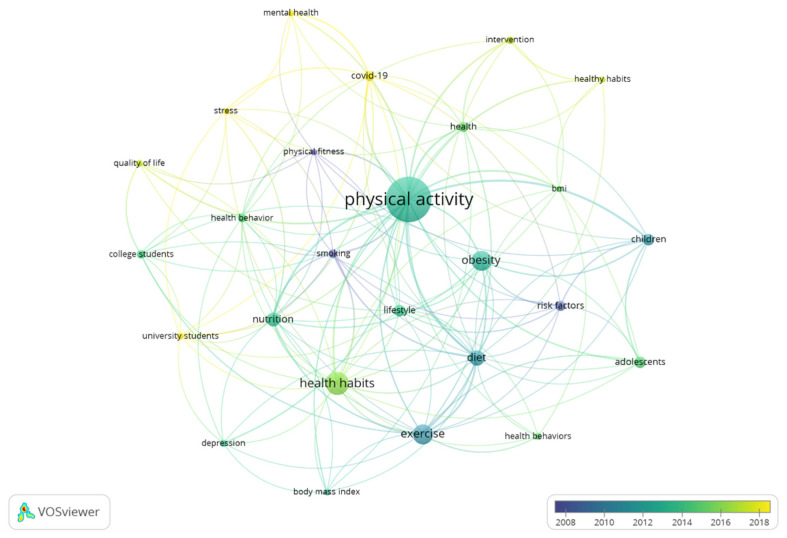
Evolution of the keywords.

In Figure 8, the interaction of two terms is shown in terms of time. The terms “stress”, “mental health”, “COVID-19”, and “university students” are the keywords that present the highest frequency in current studies. This item shows the evaluation of the terms according to the years of publication, as well as the main interests in the scientific community.

## 4. Discussion

The aim of this study was to carry out a bibliometric review to understand the state of the art in relation to physical activity and healthy habits, so that it will allow a global and reliable perspective in relation to the study object to be obtained, through a transparent and accessible model, from its origin up to the 31 December 2022. The results show the existence of 276 identified documents (262 primary studies and 14 revisions), published between the years 1990 and 2022. The number of publications has increased by 48% since 2010, and in the scientific literature, there are no documents that corroborate the results obtained, since the existing revisions are systematic or meta-analyses. Public Environmental Occupational Health, Kaprio, J., and the USA were the knowledge field, author, and country most productive, respectively. A great thematic diversity was found that related to the most used keywords by the authors, such as “physical activity”, “health habits”, “exercise”, and “obesity”. However, the bibliometric analysis allows the determination of qualitative and quantitative changes in a specific research topic [33]. For this reason, it is essential to perform bibliometric review studies, because they provide general information to researchers of physical activity and healthy habits.

The results related to WOS categories regarding the areas of knowledge show that they are reviewed by Public Environmental Occupational Health (*n* = 77), Medicine General Internal (*n* = 32), Nutrition Dietetics (*n* = 30)*,* and Sport Science (*n* = 28); these are the main areas of knowledge employed by researchers with physical activity and healthy habits. The three fields are classified in the first quartile of their thematic categories. In the scientific literature, there are no documents that corroborate the data obtained. However, research related to physical activity and healthy habits has been on the rise in recent years because of the increase in chronic illnesses [13,14,15] and because, by the year 2030, it has been shown that the majority of people will tend to be overweight or obese [1,2,3]. For this reason, it is essential to create intervention programs that aim to promote the regular practice of physical activity and healthy habits. These programs are essential to reduce childhood obesity [10], motivated by poor eating habits, reduced physical activity, and increased sedentary lifestyle, as well as the misuse of new technologies (TV, mobile devices, and video games) [10,28,29]. In this sense, the World Health Organization (WHO) has published on its official page (A healthy lifestyle—WHO recommendations), recommendations for the promotion of healthy habits. In addition, it is important to create awareness programs for families, so that they exert strong pressure on the consolidation of healthy living habits of children between six and twelve years old [51], aiming to influence better nutrition and regular involvement in physical activity.

As for the results related to the H-Index, they show the existence of 48 documents that have a large number of citations (*n* > 48). In addition, only three of the selected documents present more than 400 citations, with the three most cited manuscripts authored by Hasler et al. [48], with 481 citations, Kujala et al. [49], with 436 citations, and Vita et al. [50] with 435 citations. Therefore, analyzing the publication date of the three most cited documents, it is observed that they are from the years 1998 and 2003. They are old manuscripts. However, the authors were pioneers and got ahead of the problem of lack of regular physical activity and bad healthy lifestyles. For this reason, it is essential to increase research related to the study topic, with the aim of capturing the attention of politicians, to see that measures are implemented to improve the situation of a lack of regular physical activity and bad health habits in the population, in general. In addition, research related to physical activity and healthy habits are fields of general interest. For this reason, public institutions must create programs of habits and behaviors that generate health, since these habits will lead to the prevention of, and coping with, a disease process, becoming an active part of a person’s recovery. Therefore, it is essential to have a healthy diet, physical activity, and optimal healthy sleep.

In addition, it would be interesting to know the barriers that people have when involved in physical activity. In the scientific literature, researchers are in line with the fact that the barriers to physical activity engagement are classified into two major categories: personal (lack of time, lack of enjoyment with exercise, lack of motivation, etc.), and environmental (in-security, pollution, lack of transport, etc.) [12]. For this reason, research must be undertaken to exactly understand the barriers that prevent people from carrying out physical activity on a regular basis, as well as adopting healthy lifestyles. Therefore, it is fundamental to create strategies to promote a healthy life. Projects and initiatives for the promotion of healthy lifestyle habits should be created, such as recommendations on healthy habits in primary care or recommendations for healthy people coping with the COVID-19 pandemic.

Regarding the results related to the co-authors of the publications, there are five different working groups. The main authors are Koskenvou, Kaprio, and Sarna. In addition, these are the authors who present the greatest number of citations in relation to physical activity and healthy habits. As for the results related to the countries and regions where the investigations related to physical activity and healthy habits were carried out, they demonstrate that the main involved countries are the USA, Spain, Switzerland, and Finland. In addition, these are the countries that present the greatest number of interactions. In the same way, the USA and Finland are among the countries that receive the highest number of citations. On the other hand, depending on the year of publication, it is observed that the USA and Finland are those regions with an average year of publication that is relatively old. Likewise, Spain and Poland also have a higher number of publications, as well as the most recent ones. However, Luxembourg, Indonesia, and Portugal are regions that are increasing their number of publications, due to their current interest in the thematic object of study. In the scientific literature, there are no documents that corroborate the data obtained, because no bibliometric review relating to the object of study has been carried out. In addition, the present study provides useful information for experts who seek to know the current status related to the study object [37], as well as finding research groups with a high number of publications and connections between authors from different countries. It is also observed that to date no multidisciplinary work groups have been formed, nor relevant collaborations between groups, institutions, and countries with the purpose of increasing the productivity, effectiveness [41], and quality of the investigations related to physical activity and healthy habits. Therefore, the great interest of developed countries in promoting healthy habits in their corresponding populations can be seen. For this reason, they invest large amounts of money in investigations. In addition, it is recommended that projects to promote physical activity and healthy habits at school are carried out. In this way, people can be made aware of the importance of having optimal styles and healthy habits.

In addition, the results related to the keywords of the authors show that the most frequently used terms were “physical activity” (59), “health habits” (27), “exercise” (23), and “obesity” (23). Regarding the interaction of these terms based on temporality, the main words are “stress”, “mental health”, “COVID-19”, and “university students”. These terms are connected with the objective of the present study and the information is relevant for the authors, since it provides evidence on where future research related to physical activity and healthy habits is headed [37]. Moreover, the keywords suggest that there is little knowledge related to physical activity and healthy habits in young university students, as stressed by Rodríguez-Castellanos et al. [12]. Further, it would be interesting to increase the amount of research related to the stress of young students, in order to understand the barriers that prevent them from performing physical activity on a regular basis, as well as having good lifestyle habits. Finally, the results of this bibliometric review allow us to analyze the manuscripts in the WOS database in a general way. Therefore, future research related to physical activity and healthy habits should (a) extend this review to other databases; (b) carry out a bibliometric review that distinguishes the different contents of the manuscripts identified based on the key terms; and (c) guide research toward the use of new analysis devices in people who practice physical activity on a regular basis, since physical exercise is key to preventing chronic diseases [16,21].

The present study demonstrates the growing concern of researchers about the practice of physical activity and healthy living habits, which is a result of people assuming more sedentary behaviors in today’s society and is associated with risk behaviors (tobacco, bad eating habits, excessive use of technology, etc.), promoting behavior of greater risk to people’s health. In this sense, the practical implication of this study lies in the observation of the researchers’ concern in the conclusions of the various studies carried out in different countries, verifying a generalized need for political decision making that promotes the development of programs for the regular practice of physical activity and the promotion of healthy habits. This need must be developed with older people, to change mentalities, while also promoting the strong education of children and young people to create habits that promote their health.

## 5. Conclusions

The results of the bibliometric review related to physical activity and healthy habits show the existence of 276 documents (262 primary studies and 14 reviews) that were published between 1990 and 2022. In addition, the manuscripts with the highest number of citations were published in the fields, Public Environmental Occupational Health (*n* = 77), Medicine General Internal (*n* = 32), Nutrition Dietetics (*n* = 30), and Sport Science (*n* = 28), and were all included in the first quartile of their thematic categories. Physical activity is associated with multiple health benefits for everyone, at any age, and for both women and men. However, more and more people do not move enough, and this is due, in large part, to the fact that their lifestyles have changed toward a more sedentary model. Therefore, it is essential to create policies to promote healthy habits and styles.

Likewise, it is recommended that multidisciplinary research is carried out to establish collaborations between groups, institutions, and countries. In this way, the productivity, effectiveness, and quality of the investigations will increase, and the relations between the different countries that are investigating the object of study will increase. In addition, research will be given greater potential, and even in figures of related countries and authors, the interrelationships will be greater. Finally, this bibliometric review is a reliable and global source of knowledge that allows us to comprehensively understand the advances in research related to physical activity and healthy habits. Furthermore, it allows the interactions and collaborations between authors, research groups, and countries to be perceived.

## Figures and Tables

**Table 1:** Documents classified regarding the areas of knowledge.

Field	*n*	%
Public Environmental Occupational Health	77	27.50%
Medicine General Internal	32	11.43%
Nutrition Dietetics	30	10.71%
Sport Sciences	28	10.00%
Health Care Sciences Services	15	5.36%
Nursing	15	5.36%
Pediatrics	13	4.64%
Education Educational Research	11	3.93%
Environmental Sciences	10	3.57%
Geriatrics Gerontology	9	3.21%
Psychiatry	9	3.21%
Psychology Multidisciplinary	9	3.21%
Cardiac Cardiovascular Systems	7	2.50%
Physiology	7	2.50%
Psychology Clinical	7	2.50%
Education Scientific Disciplines	5	1.79%
Gerontology	5	1.79%
Hospitality Leisure Sport Tourism	5	1.79%
Medical Informatics	5	1.79%
Multidisciplinary Sciences	5	1.79%
Psychology Developmental	5	1.79%
Clinical Neurology	4	1.43%
Endocrinology Metabolism	4	1.43%
Neurosciences	4	1.43%
Rehabilitation	4	1.43%

## Data Availability

The data that support the findings of this study are available from the corresponding author, upon reasonable request.
